# Cytotoxicity and Genotoxicity of Ceria Nanoparticles on Different Cell Lines *in Vitro*

**DOI:** 10.3390/ijms14023065

**Published:** 2013-02-01

**Authors:** Laura De Marzi, Antonina Monaco, Joaquin De Lapuente, David Ramos, Miquel Borras, Mario Di Gioacchino, Sandro Santucci, Anna Poma

**Affiliations:** 1 Department of Life, Health and Environmental Sciences, University of L’Aquila, L’Aquila I-67100, Italy; E-Mail: laura.demarzi@univaq.it; 2 Department of Physical and Chemical Sciences, University of L’Aquila, L’Aquila I-67100, Italy; E-Mails: antonina.monaco@aquila.infn.it (A.M.); sandro.santucci@aquila.infn.it (S.S.); 3 PCB_Unit of Experimental Toxicology and Ecotoxicology (UTOX-PCB), Scientific Park of Barcelona, Barcelona 08028, Spain; E-Mails: jlapuente@pcb.ub.es (J.D.L.); dramos@pcb.ub.es (D.R.); mborras@pcb.ub.es (M.B.); 4 Immunotoxicology and Allergy Unit, Ageing Research Center, Department of Medicine and Science of Ageing, University “G. D’Annunzio” of Chieti Pescara, Chieti I-66013, Italy; E-Mail: m.digioacchino@unich.it

**Keywords:** ceria nanoparticles, short-term exposure cytotoxicity, long-term exposure cytotoxicity, genotoxicity

## Abstract

Owing to their radical scavenging and UV-filtering properties, ceria nanoparticles (CeO_2_-NPs) are currently used for various applications, including as catalysts in diesel particulate filters. Because of their ability to filter UV light, CeO_2_-NPs have garnered significant interest in the medical field and, consequently, are poised for use in various applications. The aim of this work was to investigate the effects of short-term (24 h) and long-term (10 days) CeO_2_-NP exposure to A549, CaCo2 and HepG2 cell lines. Cytotoxicity assays tested CeO_2_-NPs over a concentration range of 0.5 μg/mL to 5000 μg/mL, whereas genotoxicity assays tested CeO_2_-NPs over a concentration range of 0.5 μg/mL to 5000 μg/mL. *In vitro* assays showed almost no short-term exposure toxicity on any of the tested cell lines. Conversely, long-term CeO_2_-NP exposure proved toxic for all tested cell lines. NP genotoxicity was detectable even at 24-h exposure. HepG2 was the most sensitive cell line overall; however, the A549 line was most sensitive to the lowest concentration tested. Moreover, the results confirmed the ceria nanoparticles’ capacity to protect cells when they are exposed to well-known oxidants such as H_2_O_2_. A Comet assay was performed in the presence of both H_2_O_2_ and CeO_2_-NPs. When hydrogen peroxide was maintained at 25 μM, NPs at 0.5 μg/mL, 50 μg/mL, and 500 μg/mL protected the cells from oxidative damage. Thus, the NPs prevented H_2_O_2_-induced genotoxic damage.

## 1. Introduction

In recent years, we have participated in what seems to be a nanotechnology revolution. This revolution is characterized by an enormous increase in the production, development, and commercialization of different types of nanoparticles (NPs). The main aim is to discover the best NPs for specific technological functions. Nanomaterials (NMs) are synthesized in a variety of ways, which diversifies their nature, shape, superficial charge, dimension and the eventual coatings. Obviously, all these variables lead to nanomaterials with diverse physical and chemical properties that have charmed scientists all over the world. The applications spread from engineering to medicine and for uses ranging from electronic devices to possible uses as drug delivery systems.

Despite the many potential uses of NPs, we should remember that both humans and the environment are exposed to NMs. This statement is particularly true today, as high quantities of NPs are produced globally.

Only in recent years have institutions and the Public Health Organization taken into account the possible sources of NPs and the subsequent damages that short-term and long-term exposure to NPs can cause in both human beings and the environment. All of the *in vitro* tests performed in our study are European Commission validated assays [1,2].

CeO_2_-NPs were first commercially employed in 1999 as catalysts in diesel particulate filters to decrease the particle mass in exhaust. Envirox™, developed by Oxonica (Kidlington, UK), is an example of a CeO_2_-based borne catalyst consisting of 2% nanoparticulate cerium oxide in a mixed aliphatic/cyclo-aliphatic fluid. Envirox™ has a final CeO_2_ concentration of 5 ppm in the diesel fuel [3]. The result is a decrease in particulate and NOx emissions and an increase in the emissions of ultrafine particles, CO, hydrocarbons and volatile organic compounds [4–6]. In spite of efficiently trapping particulate matter, detailed studies have shown that cerium is emitted in the exhaust of the escaping particulate matter. Another study converted CeO_2_-NPs to a more water-soluble form(s), which potentiates increased public exposure to diesel emissions [7]. Furthermore, the presence of CeO_2_-NPs means these particles may even make it into water supplies, soil, and the food chain. Notably, a study by Lopez-Moreno *et al.* found traces of CeO_2_ in tomato, corn, cucumber and alfalfa crops [8]. Moreover, Zeyons *et al.* studied the interaction of CeO_2_-NPs with two different microorganisms: Synechocystis PCC6803 (earth’s most abundant photosynthetic cyanobacterium, which makes up a large part of the food chain’s biomass) and the RR1 strain of *E. coli* (a heterotrophic bacteria model) [9]. In addition to their use in catalytic filters, CeO_2_-NPs are industrially used in sunscreens, cosmetics and coating and surface treatments [10–12]. CeO_2_-NPs strongly absorb UV radiation and are transparent to visible light.

Notably, the cubic fluorite structure of CeO_2_-NPs also allows them to scavenge radicals. Additionally, CeO_2_-NPs tend to be non-stoichiometric compounds in which the cerium atoms can be present in both the +4 and the +3 oxidative states. These oxygen valences confer peculiar redox properties to CeO_2_-NPs. The attention to CeO_2_-NPs is now turning toward medical applications, as their radical scavenging capabilities can possibly be used to mitigate oxidative stress in different model systems [13–15]. Oxidative stress plays a principal role in different pathological conditions. For example, in ischemic heart disease, there was a direct correlation between oxidative stress and the increase of many pro-inflammatory cytokines (tumor necrosis factor-α, interleukin-I, interleukin-6, monocyte chemoattractant protein-1 and ROS) [16,17]. Notably, the levels of many antioxidants also decreased, thereby yielding favorable conditions for the initiation and/or progression of cardiac dysfunction. In particular, it is well reported that monocyte chemoattractant protein-1 (MCP-1) is strongly related to ischemic cardiomyopathy [18,19]. Niu *et al.* performed an *in vivo* study to analyze the antioxidant effects of CeO_2_-NPs on MCP-1 transgenic mice (MCP mice) by intravenously administering 15 nmol of CeO_2_-NPs to MCP mice and wild-type control mice for two weeks (two administrations/week) [20]. The results showed that CeO_2_-NPs are able to attenuate myocardial oxidative stress and inflammatory processes.

Moreover, in recent non-cellular studies performed by Linse *et al.*, CeO_2_-NPs seem to initiate protein fibril formation with β2-microglobulin, mimicking the amyloid formation mechanism involved in Alzheimer’s and Creutzfeld-Jacob’s disease [21]. These authors found that NPs are more effective at inducing protein fibrillation when compared with cognate micro-scale particles. Furthermore, NPs seem to protect hippocampal cell lines from oxidative stress [13]. Another *in vitro* study demonstrates that CeO_2_-NPs can enter into cells via a caveolin-1 and LAMP-1 endosomal compartment without invoking cytotoxic effects. Furthermore, CeO_2_-NPs can induce cellular resistance to exogenous sources of oxidative stress [22].

Despite the great promise that CeO_2_-NPs show for future medical applications and the studies aiming to evaluate their ability to exhibit antioxidant properties *in vivo*, there are still few datasets on the effects CeO_2_-NPs can have on the entire human body and on the possible reactions that uncontrolled uptake can have on human health.

Thus, it would be prudent to perform *in vitro* tests to analyze the impact of new compounds on different cell lines prior to *in vivo* testing. Cell lines are usually selected to reproduce possible modes of contact and uptake in humans; cell lines are also selected for their ability to provide possible metabolic targets. Furthermore, the European Commission created a new institute called the European Centre for the Validation of Alternative Methods (ECVAM) in October of 1991. ECVAM has the specific purpose of “developing, validating and accepting new and alternative methods that can reduce, refine or replace the use of laboratory animals” with the Directive 86/609/EEC. The “guidance manual for the testing of manufacturing nanomaterials” highlights and describes alternative methods, focusing specific attention on the importance of the 3Rs: Refinement—Reduction—Replacement [23].

In this work, the CeO_2_-NP toxicity is reported for the future comparison to ongoing *in vivo* tests. Notably, Shubert *et al.* [14] investigated the neuroprotective effects of these NPs on the HT22 hippocampal neuronal cell line, and Chen *et al.* developed a new approach to exploit the CeO_2_-NPs’ antioxidant property and to scavenge ROS formation in retinal degenerative disease [24].

The study was performed on different *in vitro* models: HepG2, human hepatic carcinoma cell line; CaCo2, cells from human colon carcinoma; and A549, a cell line from human lung carcinoma. All of these cell lines were chosen because they are commonly used in *in vitro* studies. Additionally, they are commonly employed in OECD and ECVAM toxicity testing and give an overview of potential NP exposure.

Before performing the toxicity tests, the CeO_2_-NPs were fully characterized by Fourier Transformed Infrared Spectroscopy (FTIR) and X-Ray Diffraction (XRD), which were used to determine the crystalline phases and measure the dimensions of the NPs. The NP size was confirmed by Scanning Electron Microscopy (SEM).

The biological tests yielded insight into the basal cytotoxicity and genotoxicity of the CeO_2_-NPs. To measure the cytotoxicity, we used the MTT assay and followed INVITTOX protocol n17 [25], reaching a final CeO_2_-NP concentration of 5000 μg/mL from a starting concentration of 0.5 μg/mL. The CeO_2_-NP genotoxicity was measured using the Comet assay at a final CeO_2_-NP concentration of 500 μg/mL. The DNA damage was extensive. An indirect assay was performed to evaluate the antioxidant properties of CeO_2_-NPs on *in vitro* models. This test followed the Comet assay protocol, but introduced H_2_O_2_, a well-known oxidant, along with the CeO_2_-NPs to evaluate the CeO_2_-NPs’ ability to protect cells.

## 2. Results and Discussion

### 2.1. Results

#### 2.1.1. CeO_2_-NP Characterization

**Figure 1:**
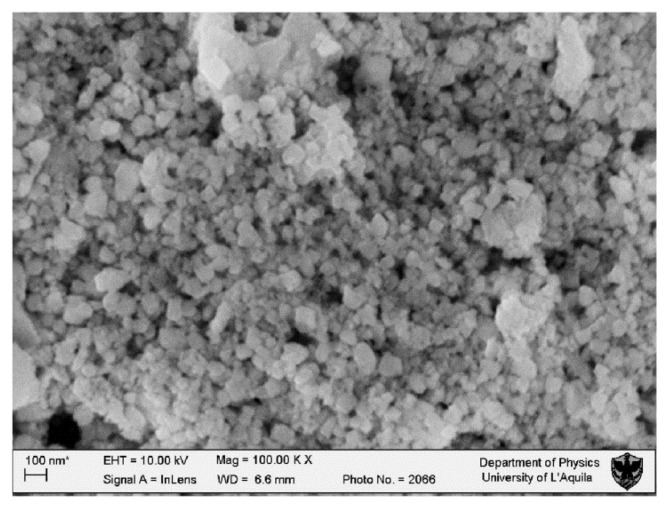
SEM imaging of nanoceria particles synthesized according to Materials and Methods; the average diameter of the nanoparticles is between 16 and 22 nm.

**Figure 2:**
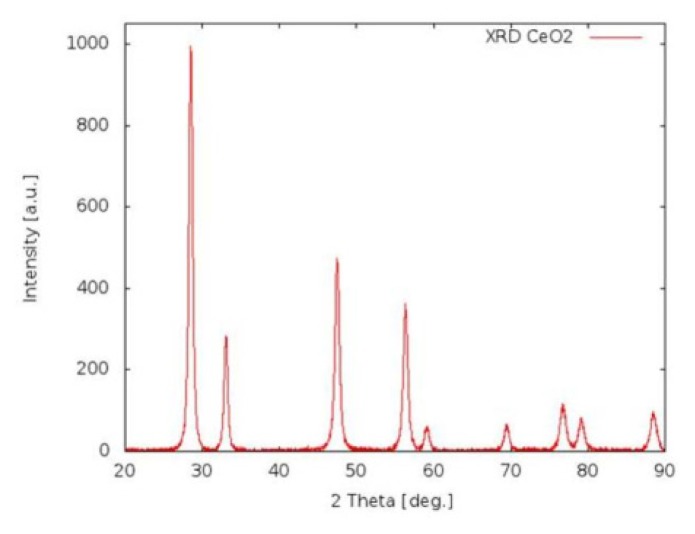
X-Ray diffraction evaluation of CeO_2_-NP crystalline phase production.

**Figure 3:**
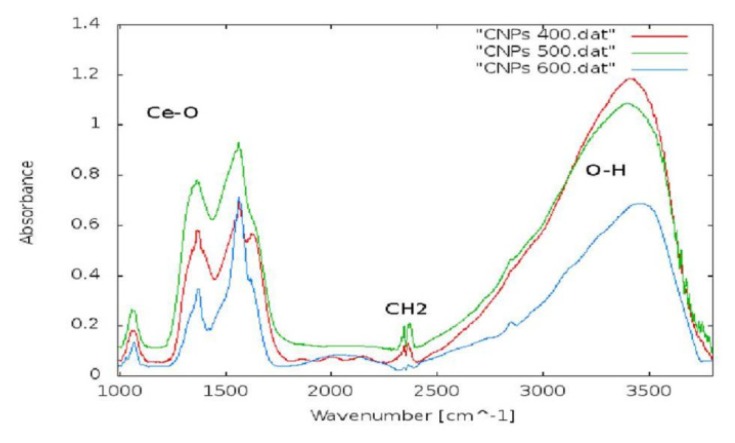
FTIR spectra: characteristic peaks of Cerium oxide (Ce–O), peaks related to residual surfactant (CH_2_), and peaks related to the water adsorbed on the sample surface are shown.

Figure 1 shows the presence of clusters approximately 40 nm in diameter. The nanoparticle surface is smooth and devoid of pores. The average nanoparticle diameter is between 16 and 22 nm, thus confirming the XRD results (Figure 2). The FTIR spectrum (Figure 3) shows characteristic frequency bands of Ce-O bond stretching, a band related to the vibrational modes of the O-H bond in water adsorbed to the sample surface and a peak ascribable to residual surfactant. A Zeta-potential of −46.2 mV in water (pH 7.4) was measured; the measured Zeta-potential indicates that dispersions are quite stable. The potential was less than −30 mV, which is commonly considered the cut-off value for stable suspensions.

#### 2.1.2. MTT Assay

**Figure 4:**
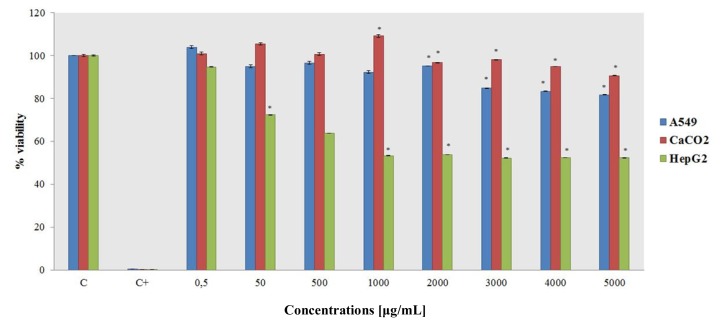
Short-term exposure cytotoxicity, as measured by the MTT assay after cells are incubated with CeO_2_–NPs for 24 h. The data are means ± SD calculated for at least three replicates for each experimental point.

**Figure 5:**
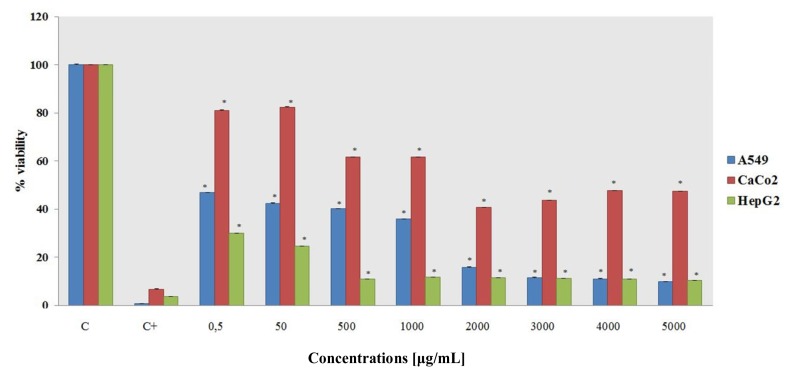
Long-term exposure cytotoxicity, as determined by the MTT assay after 10 days. The data are means ± SD calculated for at least three replicates of each experimental point. All the results are compared to the negative controls (100% viability); the positive controls showed close to 0% viability.

MTT assays over different concentrations and exposure times were used to evaluate short-term and long-term ceria nanoparticle exposure cytotoxicity. Short-term exposure toxicity was measured after 24 h, and long-term exposure toxicity was measured after 10 days. The results of the short-term and long-term exposure data vary considerably (Figure 4 and Figure 5).

Short-term exposure after 24 h shows no toxic effect in almost any of the cell lines. Only the HepG2 cell line shows a decrease in the percent viability from the 50 μg/mL concentration to the highest tested concentration.

Separately, the long-term exposure data demonstrates that CeO_2_–NPs can dramatically affect the cells’ viability. The A549 and HepG2 cell lines show a drastic decrease in the percent viability, particularly at the 2000 μg/mL concentration. CaCo2 is the only exception, as it seems to be only partially affected by CeO_2_–NPs.

#### 2.1.3. Alkaline Comet Assay

**Figure 6:**
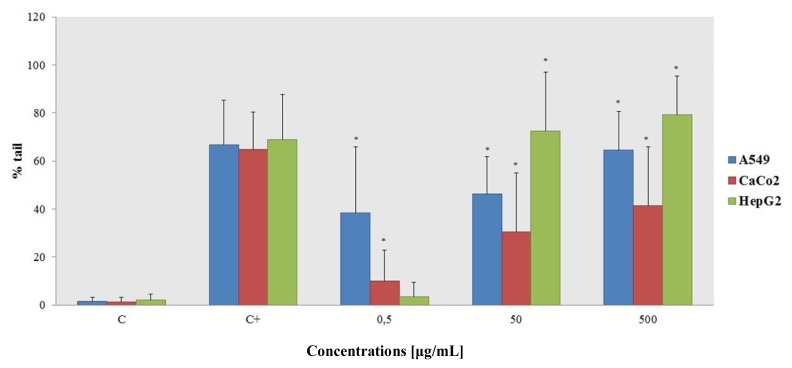
Comet assay. The results are compared to the negative control and are the means ± SD calculated for at least three replicates for each experimental point.

The comet head contains undamaged DNA, and the extent of damage is assessed by the percentage of DNA in the tail. The results shown in Figure 6 demonstrate DNA damage in the presence of the cerium oxide nanoparticles. At the highest concentration tested, comet formation was comparable to the positive control.

The NP genotoxic effect is strictly dose dependent, and HepG2 is the most sensitive cell line, providing a convenient and sensitive tool for the rapid screening of nanomaterial samples with potentially genotoxic and cytotoxic effects. The liver is of particular importance to toxicological research; therefore, using *in vitro* hepatic systems for nanotoxicity studies may garner increased attention.

An unexpected result was obtained on the A549 cell line—at the lowest concentration tested, it appeared to be the most sensitive cell line.

**Figure 7:**
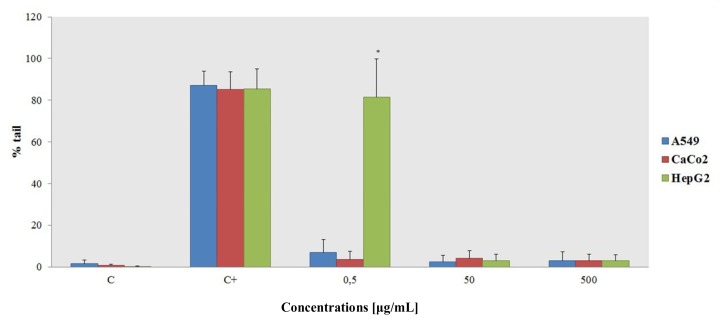
The Comet assay after the addition of 25 μM H_2_O_2_ to evaluate the protective ability of CeO_2_–NPs. The results are compared to the negative control and are the means ± SD for at least 3 replicates for each experimental point.

It is well known that CeO_2_-NPs have the ability to scavenge radicals, but the available literature focuses mostly on *in vivo* experiments. After the addition of 25 μM H_2_O_2_ to the cells in the presence of CeO_2_-NPs, the Comet test (Figure 7) showed that the presence of comets at all concentrations tested is comparable to the negative control, notwithstanding an anomalous result for the HepG2 cells at the lowest tested concentration. Presumably, the increase in NP concentration effectively protects the cells by scavenging free radicals in the cells’ environment. This peculiar behavior can be explained by the chemical nature of the NPs—ROS in the medium react preferentially with the NPs, but also with six cell types and DNA.

### 2.2. Discussion

The effects of short-term (24 h) and long-term (10 days) exposure of different cell lines to ceria nanoparticles was investigated. Specifically, the effects of ceria nanoparticles on the cytotoxicity and genotoxicity of three cell lines, A549, CaCo2 and HepG2, were tested over a range of nanoparticle concentrations (0.5 μg/mL to 5000 μg/mL). Additionally, the *in vitro* antioxidant effects of the ceria nanoparticles were investigated.

CeO_2_–NPs’ ability to scavenge radicals has garnered significant scientific attention, as they may be beneficial in treating degenerative and neuronal diseases.

Despite the great promise that CeO_2_–NPs show especially for future medical applications and the extensive *in vivo* testing in mouse models, few studies test how CeO_2_–NPs react in the human body. Likewise, few studies explore the possible reactions that uncontrolled uptake can have on human health or comparatively evaluate *in vitro* toxicity on different normal and cancer cell lines.

We analyzed the 24-h exposure toxicity, which showed no toxic effects on the cell lines employed in this study. Separately, the long-term exposure toxicity (10-day treatment) as measured by MTT assays demonstrated that CeO_2_–NPs can drastically affect the cells’ viability. The A549 and HepG2 lines had a drastic decrease in the percent viability at a nanoparticle concentration of 50 μg/mL, while the CaCo2 line seems to be only partially affected by ceria NPs. These results may be useful for future cerium oxide particle applications in the treatment of tumor cells.

The Comet assay showed how CeO_2_–NPs can induce genotoxic damage. The most sensitive cell line overall was the HepG2 line, but the A549 line was the most sensitive at the lowest concentration tested. The cerium oxide genotoxicity data are conflicting, and results are largely dependent on the cellular system tested. For example, Pierscionek *et al.* measured the sister chromatid exchanges and the DNA damage (using an alkaline Comet assay) of cultured human lens epithelial cells exposed to 5 or 10 μg/mL of CeO_2_–NPs [26]. Nanoceria at these dosages did not cause any DNA damage or significantly increase the number of sister chromatid exchanges. The absence of genotoxic effects on lens cells suggests that nanoceria, in the doses and exposures tested in that study, are not deleterious to the eye lens.

The assays in the present work were performed to establish correlations between the *in vivo* results from mouse models and *in vitro* analyses. The antioxidant activity of CeO_2_–NPs has been especially highlighted by *in vivo* experiments in the recent literature [13,20,21]. In particular, the second type of Comet assay is based on the idea that genotoxic damage by a well-known oxidant (H_2_O_2_ in this example) should be mitigated by the presence CeO_2_–NPs. An increase in the NP concentration while the hydrogen peroxide concentration remains constant effectively protects cells from oxidative damage.

## 3. Experimental Section

### 3.1. Nanoparticle Preparation and Characterization

One hundred grams of ethylene glycol (surfactant) were placed in a glass beaker with 6 g of Ce(NO_3_)_3_·6H_2_O (precursor, purity: 99.5%).

The solution was placed on a magnetic stirrer at room temperature for half an hour until the precursor was completely dissolved. Then, drops of NH_4_OH were added until the color of the solution changed from transparent to orange. The beaker was covered and placed in a bigger beaker containing water at a temperature of 50 °C.

The system was placed on a magnetic stirrer and heating block with a set temperature of 50 °C and left there for 14 h. Then, the slurry, which again changed its color from orange to red amber, was placed in ceramic crucibles and was calcinated in an oven under ambient conditions.

The CeO_2_–NPs were studied by X-Ray Diffraction (or XRD) and Fourier Transformed Infrared Spectroscopy (FTIR) to establish their crystalline phase formation and dimensions. FTIR spectroscopy was collected with a Perkin Elmer spectrum BX spectrometer. X-Ray Diffraction (XRD) was carried out using a diffractometer made up of a Siemens D5000 X-ray source, a monochromator and focuser, a carrier plate, and a detector of the diffracted radiation. Scanning Electron Microscopy (or SEM) measurements were carried out to evaluate the shape, dimensions and agglomerates of the NPs. SEM observations were made using NPs dispersed in culture medium. SEM images were acquired using a LEO GEMINI 1530 model microscope interfaced to a PC through ZEISS, an image processing software.

The surface charge of the NPs was measured by Dynamic Light Scattering (DLS, Zetasizer Nano-ZS, Malvern Instruments Ltd., UK). Samples for the measurements of the zeta-potential were prepared by dispersing 10 g of material in 10 mL of distilled water.

### 3.2. Cell Culture Maintenance

A549 (human alveolar adenocarcinoma cell line, CCL-185), CaCo2 (human colorectal adenocarcinoma cell lines, HTB-37), HepG_2_ (human hepatic carcinoma cell line, HB-8065) and Balb/3T3 (mouse fibroblast cell line, CCL-163) cell lines were used for the *in vitro* assays. The cells were maintained in a Dulbecco’s Modified Eagle Medium (DMEM) culture medium replete with 10% Inactivated Fetal Bovine serum (HyClone). Additionally 1% l-glutamine (Sigma-Aldrich, St. Louis, MO, USA) was added for the A549, HepG_2_ and Balb/3T3 cell lines. l-glutamine to a final concentration of 2% was added to the CaCo2 cell line. Finally, 0.5% Penicillin/Streptomycin was added to the media for all cell lines (Sigma-Aldrich, St. Louis, MO, USA). The cells were always incubated (Thermo Forma Incubator-HEPA filters) at a temperature of 37 °C in an atmosphere containing 5% CO_2_.

### 3.3. MTT Assay

The MTT assay was performed on A549, CaCo2, HepG_2_ and Balb/3T3 cell lines according to protocol 17 of INVITTOX (Ecvam). We seeded approximately 1 × 10^3^ cells in 200 μL of media in each well of a 96-well plate (Nunc). The day after seeding, we applied the CeO_2_–NPs to the following final concentrations in the culture medium: 0.5 μg/mL, 50 μg/mL, 500 μg/mL, 1000 μg/mL, 2000 μg/mL, 3000 μg/mL, 4000 μg/mL, and 5000 μg/mL. Negative control proliferation was detected the day after starting the culture. The positive control used 1 mM 5-Fluorouracil (5-FU) in the 24-h and 10-day exposure tests. The cells were exposed to the CeO_2_–NPs for 24 h or 10 days to evaluate their short-term exposure and long-term exposure toxicity, respectively. In the 10-day exposure procedure, we changed the culture medium after 5 days. In both cases, at the end of the exposure period, we added 20 μL of MTT to each well. After a 2-h incubation, we eliminated the medium using the MTT and added 130 μL of desorb solution (96% of 2-propanol and 0.7% of SDS). The assay was performed at a wavelength of 540 nm (Bio-Tek Instruments spectrophotometer, Power Wave X, Winooski, VT, USA).

### 3.4. Alkaline Comet Assay

The comet assay was performed on A549, CaCo2 and HepG_2_ cell lines according to Singh *et al.* [27]. The seeding was made using 10^4^ cells on 100 μL of complete culture medium per well in a 96-well-plate format. The day after seeding the cells, we treated them with CeO_2_–NPs using the following concentrations: 0.5 μg/mL, 50 μg/mL, and 500 μg/mL. We also treated the negative and positive controls (metilmetane sulfonate 1:100 *v*/*v* in water). The exposure time was 24 h. At the end of the exposure period, we collected the cells through trypsinization, followed by centrifugation at 1100 rpm for one minute to obtain the pellet and avoid cell loss. After the centrifugations, we eliminated the supernatant and resuspended the pellet in 140 μL of 0.9% agarose in milliQ water (low-melting point agarose—Sigma Aldrich, St. Louis, MO, USA). The suspensions of cells in agarose were then applied dropwise to microscope slides containing an agarose layer (agarose electrophoresis grade—Invitrogen prepared with a 1% concentration in milliQ water), put in a freezer for 10 min and then layered in the lysis buffer for 1 h. The lysis buffer was freshly prepared with 2.5 M NaCl, 100 mM Na_2_EDTA and 10 mM Tris in milliQ water. The final pH was adjusted to 10 and maintained at 4 °C. Lysis was performed at 4 °C. After the lysis exposure, the samples are left for 30 min in the electrophoretic buffer, and the samples were electrophoresed for 40 min. The electrophoresis buffer is prepared, with 1 mM Na_2_EDTA and 300 mM NaOH in milliQ water at a temperature of 4 °C. Prior to use, the buffer pH is measured to ensure that it is greater than 13. At the end of the electrophoretic run, the samples are put in a neutralizing buffer (1 M Tris, milliQ water, with a pH of 7.5) three times for 5 min each time. The samples are then stained with 4′,6-diamidino-2-phenylindole (DAPI) and observed using a fluorescent microscope (Nikon Eclipse E600 with super high pressure mercury lamp). Sixty randomly selected cells from each concentration tested were scored using Comet IV image analysis software. The fluorescence microscope is a Nikon Eclipse E400, Japan. The analysis was performed measuring the tail moment (tail length × fraction of DNA in the tail) and comparing the different concentrations with the 10% solvent control.

We further used H_2_O_2_ (Sigma-Aldrich, St. Louis, MO, USA) as positive control, as it is a well-known oxidant that can produce breaks in DNA. We used the same NP concentrations tested in the Comet assay (0.5 μg/mL, 50 μg/mL, and 500 μg/mL) and applied a genotoxic amount of H_2_O_2_ (25 μM) to all the wells except the negative control ones. After a 10-min incubation with H_2_O_2_, we trypsinized the cells and proceeded with the normal Comet assay protocol.

### 3.5. Statistical Treatment of the Data

The means and standard deviations (SD) were calculated for descriptive statistical documentation. The data are the means ± SD calculated for at least three replicates for each experimental point. Student’s t-test was applied for analytical statistics.

## 4. Conclusions

The results showed that the CeO_2_–NPs have high long-term exposure toxicity but almost no short-term exposure toxicity on all the cell lines tested. Evaluating the NP genotoxicity showed that the positive result at 24-h exposure may be due to ROS damages induced by the NPs themselves. Notably, NPs can induce genotoxic damage to the cell when present alone in the cell culture medium but can, conversely, protect the cells from oxidative stress when another genotoxic compound is also present. However, more studies are also necessary to distinguish between specific hypotheses regarding the mechanism of interaction between CeO_2_–NPs and cells [28–30]. The cyto- and genotoxicity of CeO_2_–NPs are safety concerns for *in vivo* applications. In this regard, we are conducting experiments to test the *in vivo* effects after multi-dose administrations of CeO_2_-NPs and to determine whether there is any accumulation of CeO_2_–NPs in specific organs.
